# Supplementary material for: Bridgin connects the outer kinetochore to centromeric chromatin
Source: Nat Commun. 2021 Jan 8;12:146. doi: 10.1038/s41467-020-20161-9 (PMC7794384; doi:10.1038/s41467-020-20161-9)
Supplement: Supplementary file 1 — Supplementary Information [file 41467_2020_20161_MOESM1_ESM.pdf]

Supplementary information for

Bridgin connects the outer kinetochore to centromeric chromatin

by

Sridhar *et al.*

This PDF file includes:

Supplementary Figures 1-8

Supplementary Tables 1-5

Supplementary References

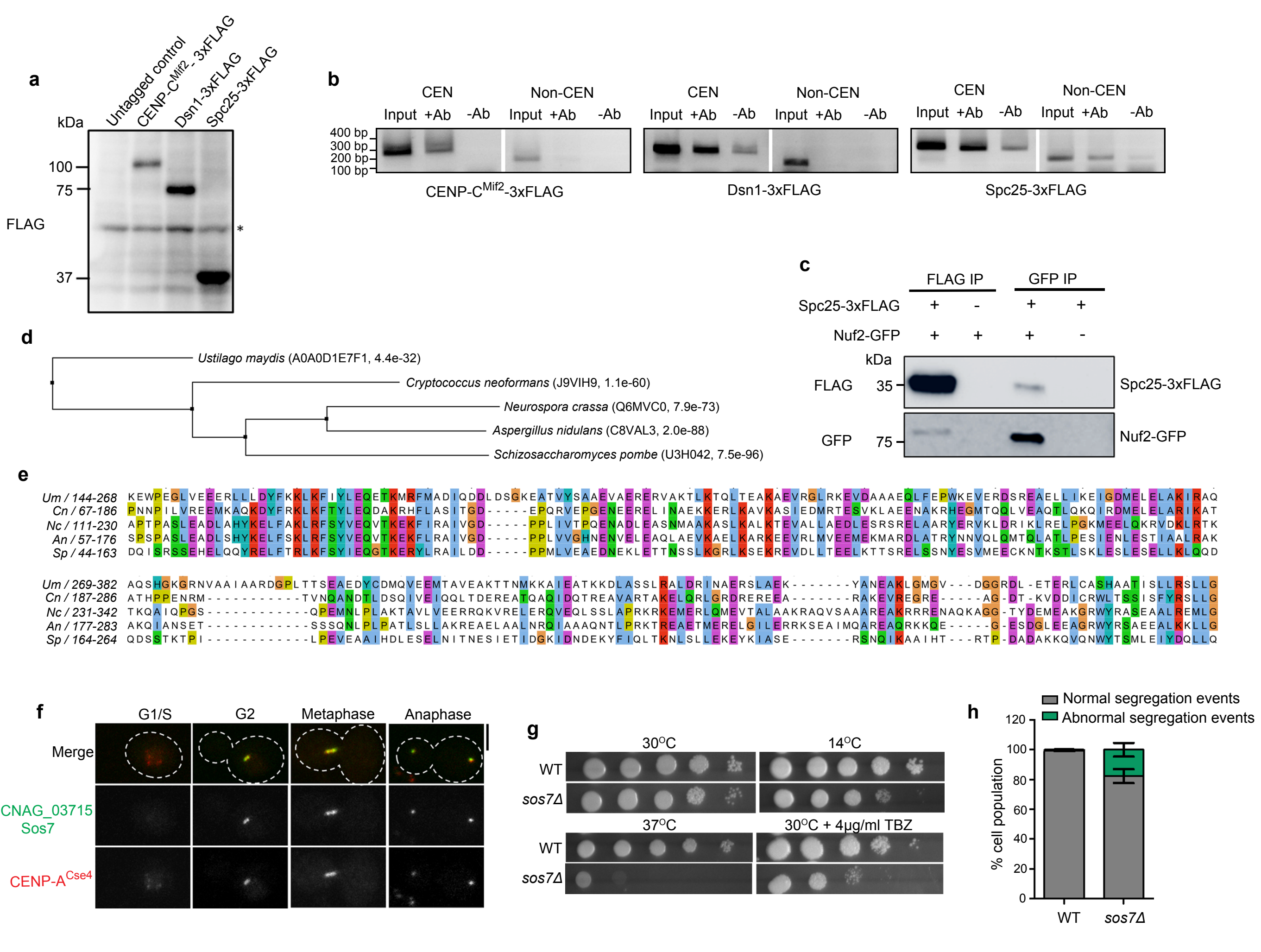

i

Proteins of unknown function

ORF	Protein	WT control		CENP-C Mif2 -3xFLAG		Dsn1 -3xFLAG		Spc25 -3xFLAG		Domains present	Domain function
		%C	TSC	%C	TSC	%C	TSC	%C	TSC		
CNAG_01903	Bkt1	-	-	11	9	43	120	44	212	FHA domain	Phosphopeptide recognition
CNAG_03959	Bkt2	6	1	53	17	65	13	72	20	-	-
CNAG_02701	Bkt3	-	-	4	2	16	10	12	6	BAG domain	Modulators of chaperone activity

j

Predicted chromatin binding proteins with no known function at the kinetochore

ORF	Protein	WT control		CENP-C Mif2- 3xFLAG		Dsn1 -3xFLAG		Spc25 -3xFLAG		Domains present
		%C	TSC	%C	TSC	%C	TSC	%C	TSC	
CNAG_01340	Yta7	-	-	14	32	30	177	10	14	ATPase, Bromo-domain
CNAG_03962	Mcm6	-	-	12	13	36	51	14	15	DNA helicase

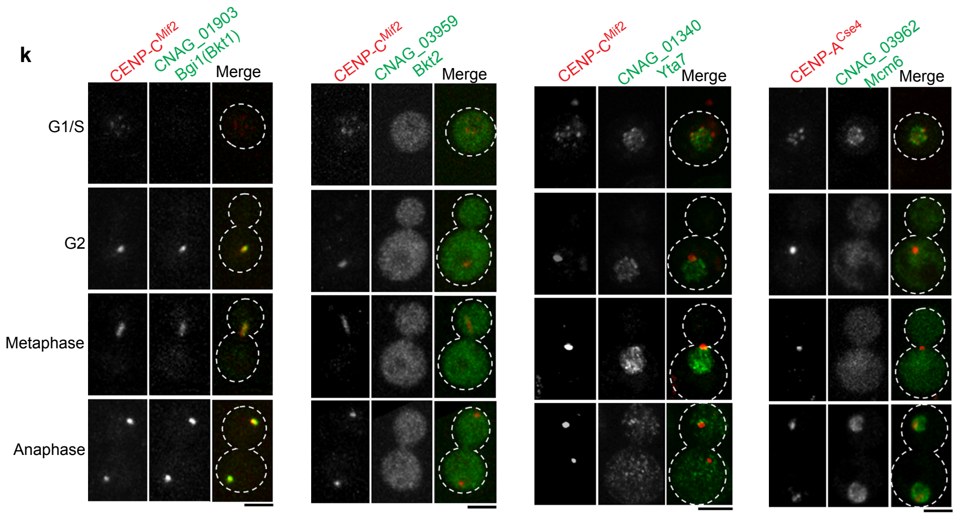

Supplementary Figure 1

Supplementary figure 1. Screening of identified kinetochore interacting proteins in *C. neoformans*. a Immunoblot analysis of native 3xFLAG tagged kinetochore proteins CENP-C^{Mif2} in SHR896, Dsn1 in SHR824, Spc25 in SHR823 and untagged control H99 strains after thiabendazole treatment (enriching for cells in M phase). Asterix represents a non-specific band. **b** Functional validation of 3xFLAG tagged kinetochore proteins mentioned in **(a)** by ChIP-polymerase chain reaction (PCR). **c** Coimmunoprecipitation (co-IP) validation of Spc25 and Nuf2 interaction. Nuf2-GFP and Spc25-3xFLAG in SHR823 were independently immunoprecipitated to score for their interaction with each other. Spc25 expressing SHR861 and Nuf2 expressing SHR516 was used as control. **d** Homologs of Sos7 in the mentioned fungal species were identified by using the previously described *Schizosaccharomyces pombe* homolog (U3H042). The identified homologs and their confidence score are mentioned next to the respective species. A cladogram describing the relationship between the identified homologs is shown. **e** Alignment of the homologs identified in **(d)** was performed using T-Coffee and visualized using Jalview. **f** Sos7 (Knl1C) in SHR845 was visualized across cell-cycle stages. mCherry-CENP-A^{Cse4} marks the kinetochore. Scale bar, 3 μm. **g** Serial 10-fold dilutions starting from $2x10^5$ cells were spotted on indicated plates for wild-type (WT) control (CNVY108, *SOS7*) and sos7 null-mutant (SHR835, *sos7Δ::NEO*). *sos7Δ* was part of the spotting perform on plates described in **Fig. 4g**, thus share the WT parental control panel histone GFP-H4. **h** Phenotypes of segregation events were scored at 30˚C using the nuclear marker histone GFP-H4 in wild-type control (CNVY108) and the Sos7 null-mutant (SHR835). The data represents the mean ± S.D. of four independent experiments. **i** List of common interacting proteins identified with no known function. These protein hits showed conservation across basidiomycetes and were identified in CENP-C^{Mif2}, Dsn1, and Spc25 FLAG affinity-purified eluates described in **(Fig. 1c)**. Predicted domains and domain functions are listed. **j** List of proteins identified as interacting partners in CENP-C^{Mif2}, Dsn1, and Spc25 FLAG affinity purifications described in **(Fig. 1c)** known to bind chromatin but with no report of a strong association with kinetochore proteins. **(i-j)** Percent coverage (%C) and total spectrum count (TSC) are indicated. **k** Localization of carboxy-terminus tagged V5-GFP constructs across mentioned interphase and mitotic stages. CENP-C^{Mif2}-mCherry for $Bgi1^{Bkt1}$ in SHR876, Bkt2 in SHR897 and Yta7 in SHR842 or mCherry-CENP-A^{Cse4} for Mcm6 in SHR905 marks the kinetochores. Scale bar, 3 µm. Source data are available as a Source Data file.

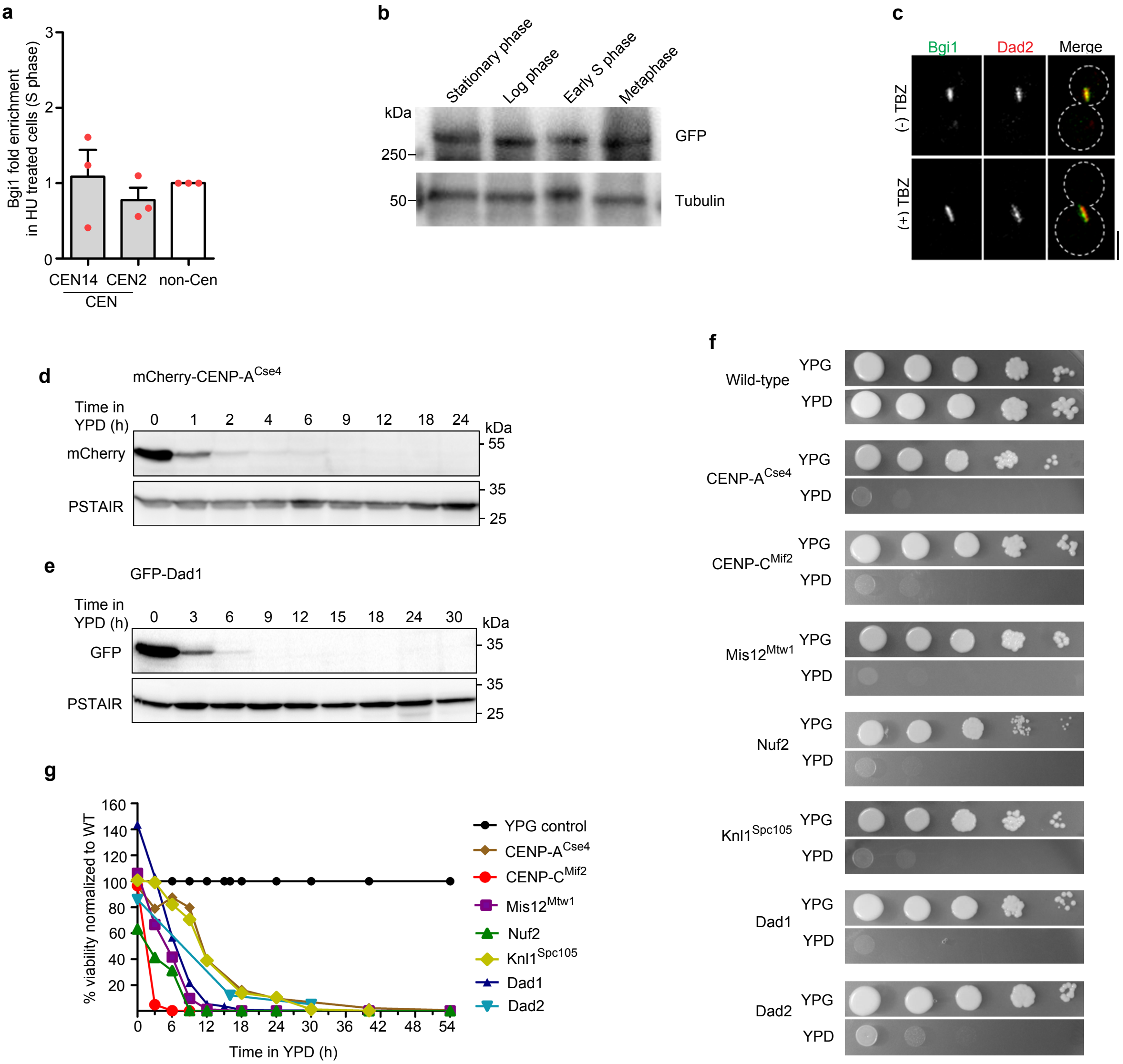

Supplementary Figure 2

Supplementary figure 2. Bridgin recruitment to the kinetochore in G2/M is not a consequence of regulated protein expression or spindle integrity. a Measurement of Bgi1-V5-GFP levels at the kinetochore at early S-phase (200 mM hydroxyurea [HU] treatment for 3 h) in SHR870 cells by cross-linked ChIP-qPCR. The data represents the mean ± standard deviation (S.D.) of three independent experiments. **(Fig. 2a)** represents the location of the qPCR primers used. **b** The cellular pool of bridgin in SHR870 across various cell cycle stages was assessed using immuno-blot analysis. **c** Representative images of Bgi1 co-localized with Dad2 in strain SHR909 (*BGI1::BGI1-V5-GFP, DAD2::DAD2-mCherry*) with and without 10 µg/ml thiabendazole (TBZ) treatment for 3 h. Scale bar, 3 µm. **d and e** Total cellular protein pool of **(d)** CENP-A^{Cse4} and **(e)** Dad1 in their respective conditional mutant strains of SHR702 and SHR710 were determined over time following growth in non-permissive (YPD, -) media by immuno-blot analysis. **f** Conditional kinetochore mutants of CENP-A^{Cse4} (SHR702, *CENP-A^{CSE4}::GAL7p-mCherry-CENP-A^{CSE4}*), CENP-C^{Mif2} (SHR716, *CENP-C^{MIF2}::GAL7p-GFP-CENP-C^{MIF2}*), $Mis12^{Mtw1}$ (SHR736, *$MIS12^{MTW1}$:: GAL7p-mCherry-$MIS12^{MTW1}$*), Nuf2 (SHR718, *NUF2::GAL7p-GFP-NUF2*), $Knl1^{Spc105}$ (SHR807, *$KNL1^{SPC105}$::GAL7p-GFP-$KNL1^{SPC105}$*), Dad1 (SHR710, *DAD1::GAL7p-GFP-DAD1*), Dad2 (SHR738, *DAD2::GAL7p-mCherry-DAD2*) and wild-type control (H99) were serially diluted 10-fold starting from $2x10^5$ cells and spotted on permissive (YPG,+) and non-permissive (YPD, -) plates. **g** Percent viability of the kinetochore conditional mutants mentioned in **(f)** was calculated over time upon growth in the non-permissive (YPD, -) condition. Percent viability obtained for YPG controls was normalized to 100. The data represents the mean ± S.D. of two independent experiments. Source data are available as a Source Data file.

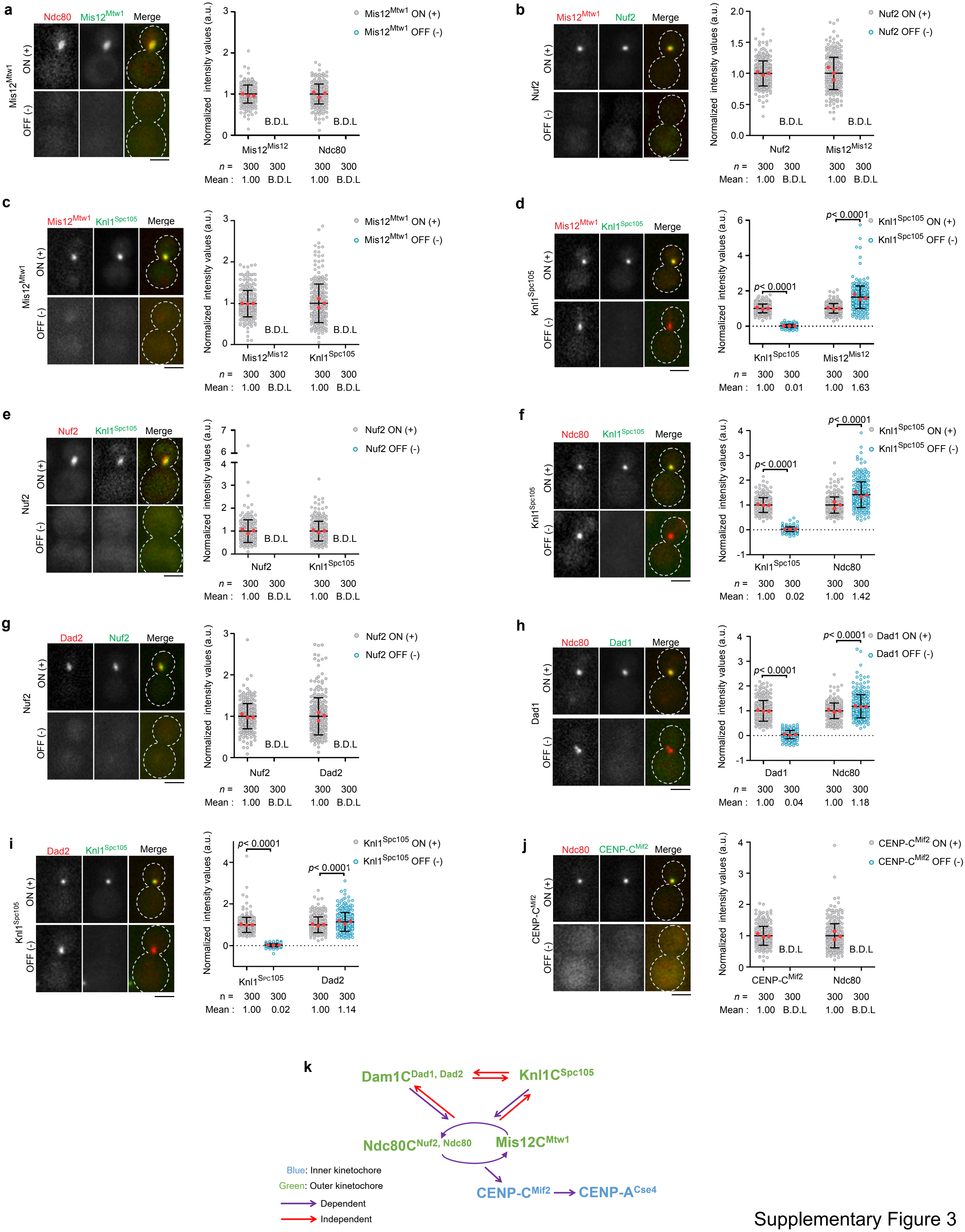

Supplementary Figure 3

Supplementary figure 3. Interdependencies of protein sub-complexes at the *C. neoformans* kinetochore. a-j Microscopic evaluation of interdependencies amongst kinetochore sub-complexes. As described in **Fig. 2b** a test kinetochore protein was fluorescently tagged in the strain background of a kinetochore conditional mutant. Signal intensities of the test protein and the fluorescently tagged conditional mutant levels were measured under permissive (+) or non-permissive (-) conditions of the *GAL7* promoter. Representative images and signal measurements depict the phenotype in non-permissive conditions after 6 h for CENP-C^{Mif2}, 12 h for $Mis12^{Mtw1}$, 15 h for Nuf2, 18 h for $Knl1^{Spc105}$, 9 h for Dad1 and 18 h for Dad2 conditional mutants. Under conditions of strong influence of the conditional mutant on the localization of the test protein, kinetochore signals were below detectable levels (B.D.L), as mentioned. *Left*, Representative micrograph of the fluorescently tagged test and conditional kinetochore proteins in the permissive (+) or non-permissive (-) conditions of the *GAL7* promoter. Scale bar, 3 µm. *Right*, fluorescent intensity measurements of the tagged test and conditional kinetochore proteins in the permissive (+) or non-permissive (-) conditions of the *GAL7* promoter were performed and normalized to their respective mean signals in the permissive condition. The data represents the results of three independent experiments with 100 cells each. The red dot represents the mean of one experiment, mean ± S.D. are shown. For statistical comparison of differences between the samples Mann-Whitney two-tailed analysis was applied, *p*-values show significant differences. The described interdependencies are : **(a)** Ndc80 (Ndc80C) on $Mis12^{Mtw1}$ (Mis12C) using strain SHR724 (*NDC80::NDC80-mCherry,* $MIS12^{MTW1}$*::GAL7p-GFP-*$MIS12^{MTW1}$). **(b)** $Mis12^{Mtw1}$ (Mis12C) on Nuf2 (Ndc80C) using strain SHR732 ($MIS12^{MTW1}$*::*$MIS12^{MTW1}$*-mCherry, NUF2::GAL7p-GFP-NUF2*). **(c)** $Knl1^{Spc105}$ (Knl1C) on $Mis12^{Mtw1}$ (Mis12C) using strain SHR768 ($KNL1^{SPC105}$*::*$KNL1^{SPC105}$*-GFP,* $MIS12^{MTW1}$*::GAL7p-mCherry-*$MIS12^{MTW1}$). **(d)** $Mis12^{Mtw1}$ (Mis12C) on $Knl1^{Spc105}$ (Knl1C) using strain SHR771 ($MIS12^{MTW1}$*::*$MIS12^{MTW1}$*-mCherry,* $KNL1^{SPC105}$*::GAL7p-GFP-*$KNL1^{SPC105}$). **(e)** $Knl1^{Spc105}$ (Knl1C) on Nuf2 (Ndc80C) using strain SHR910 ($KNL1^{SPC105}$*::*$KNL1^{SPC105}$*-GFP, NUF2::GAL7p-mCherry-NUF2*). **(f)** Ndc80 (Ndc80C) on $Knl1^{Spc105}$ (Knl1C) using strain SHR798 (*NDC80::NDC80-mCherry,* $KNL1^{SPC105}$*::GAL7p-GFP-*$KNL1^{SPC105}$). **(g)** Dad2 (Dam1C) on Nuf2 (Ndc80C) using strain SHR911 (*DAD2::DAD2-mCherry, NUF2::GAL7p-GFP-NUF2*). **(h)** Ndc80 (Ndc80C) on Dad1 (Dam1C) using strain SHR713 (*NDC80::NDC80-mCherry, DAD1::GAL7p-GFP-DAD1*). **(i)** Dad2 (Dam1C) on $Knl1^{Spc105}$ (Knl1C) using strain SHR788 (*DAD2::DAD2-mCherry, GAL7p-GFP-*$KNL1^{SPC105}$). **(j)** Ndc80 (Ndc80C) on CENP-C^{Mif2} using strain SHR720 (*NDC80::NDC80-mCherry, CENP-*C^{MIF2}*::GAL7p-GFP-CENP-*C^{MIF2}). **k** Schematic of interdependencies observed across kinetochore sub-complexes at the *C. neoformans* kinetochore. Source data are available as a Source Data file.

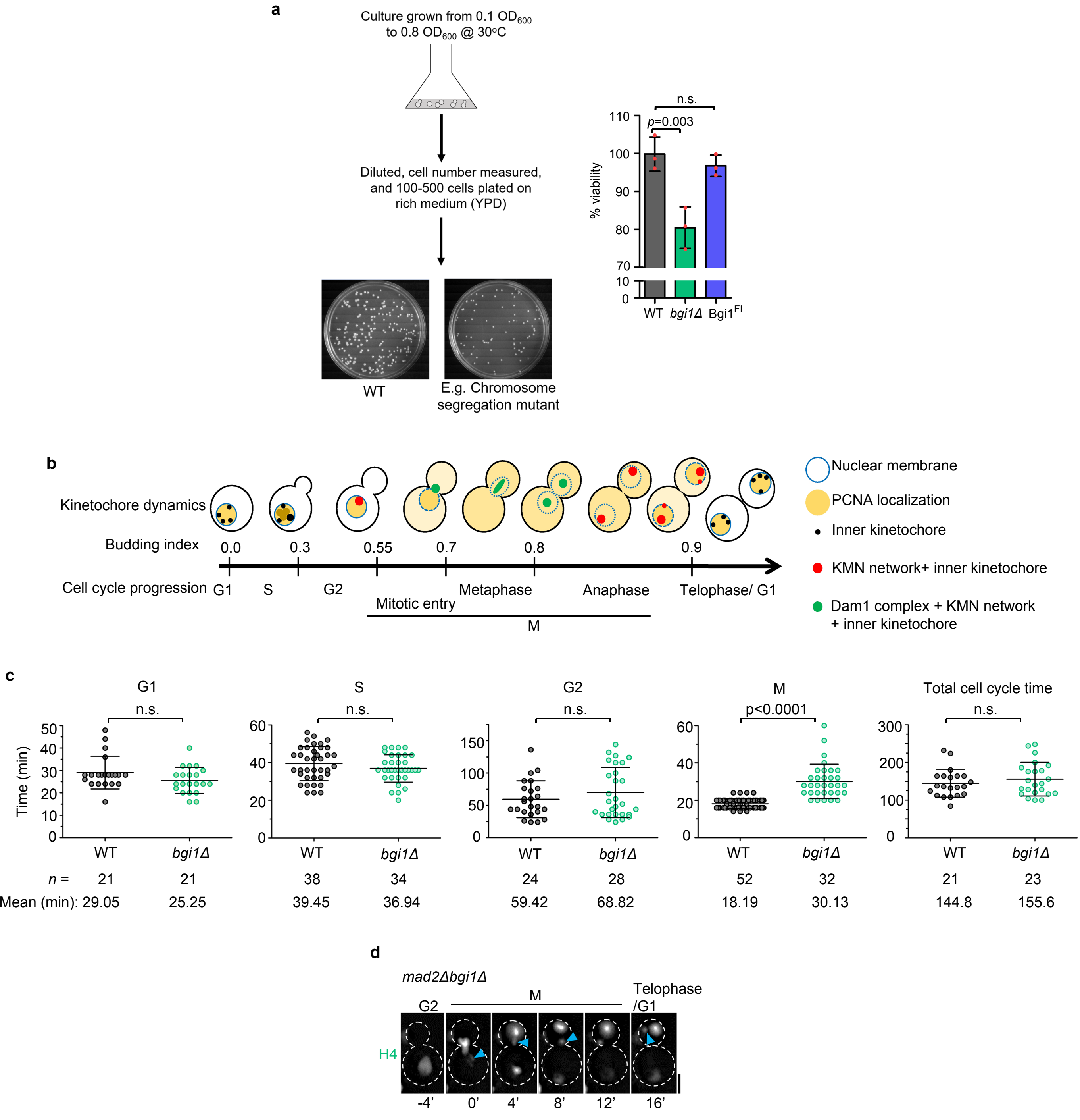

Supplementary Figure 4

Supplementary figure 4. Loss of bridgin triggers missegregation of chromosomes resulting in reduced cell viability in *C. neoformans*. a *Left*, schematic of experimental design to estimate cell viability. *Right*, colony-forming units (CFU) were counted after 48 h at 30°C and tabulated in wild-type control (CNVY121), bridgin null-mutant (SHR832) and Bgi1^{FL} complemented strain (SHR879). The data represents the mean ± S.D. of three independent experiments. One-way ANOVA test followed by Dunn's multiple comparison test was used to calculate the statistical significance of differences (the *p*-values show the difference compared to wild-type, ns: non-significant). **b** Graphical summary of cell cycle markers used to determine cell cycle stages in *C. neoformans*[1,2]. Histone H4 tagged strains were used where other markers were unavailable to determine stages of M phase. Budding index mentioned is an approximate of daughter bud: mother bud diameter ratio observed in log phase growing cells. **c** Comparison of time spent by wild-type control (SHR854) and bridgin null-mutant (SHR873) in distinct cell cycle stages. Cell cycle stages were determined at 30˚C using GFP-PCNA and histone H4-mCherry as described in **(b)**. Mean ± S.D. is indicated. *n* indicates the number of live-cells measured. Each dot represents a single live-cell measurement. For statistical comparison of differences between the samples Mann-Whitney two-tailed analysis was applied, *p*-values show significant differences. **d** Representative time-lapse of *mad2Δ bgi1Δ* double mutant (SHR866) depicting the formation of micronuclei, in the daughter cell. Blue arrows point to an initial unattached chromosome that results in a micronuclei formation. Scale, 3 µm. Source data are available as a Source Data file.

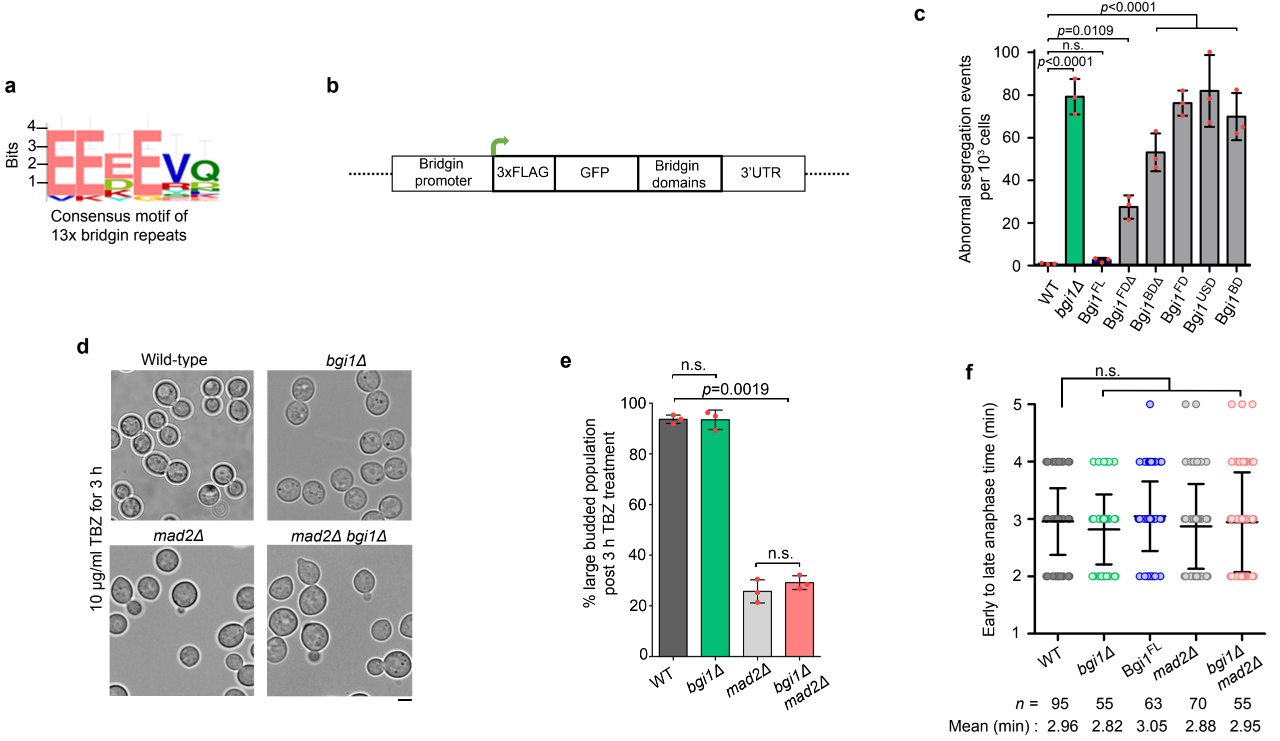

Supplementary Figure 5

Supplementary figure 5. Bridgin does not influence spindle assembly checkpoint (SAC) activity and spindle dynamics. a A motif was identified using MEME suit with the alignment of the 13 bridgin repeats of 8-amino acid in length. **b** Schematic of the bridgin domain deletion constructs. **c** Rate of abnormal segregation was measured at 30˚C using histone H4-mCherry for strains as mentioned in **(Fig. 5e)** which include wild-type control (CNVY121), bridgin null mutant (SHR832), reintegration strains of $Bgi1^{FL}$ (SHR879), $Bgi1^{FD\Delta}$ (SHR913), $Bgi1^{BD\Delta}$ (SHR880), $Bgi1^{FD}$ (SHR915), $Bgi1^{USD}$ (SHR916), and $Bgi1^{BD}$ (SHR917) and normalized to events per 10^3. The data represents the mean ± S.D. of three independent experiments. One-way ANOVA test with Dunnett's multiple comparisons test was used to calculate the statistical significance of differences (*p*-values compared to wild-type control). **d and e** Measurement of SAC activity. Wild-type control (CNVY121), *bgi1Δ* (SHR832), *mad2Δ* (SHR741) and the *mad2Δ bgi1Δ* double mutant (SHR866) cells were treated for 3 h with 10 µg/ml of TBZ. **(d)** Representative bright-field micrographs. Scale bar, 3 µm. **(e)** Percent large budded cell population was determined by scoring for cells with a budding index of >0.55. The data represents the mean ± S.D. of three independent experiments. One-way ANOVA test with Dunnett's multiple comparisons test was used to calculate the statistical significance of differences (*p*-values compared to respective controls of wild-type and *mad2Δ*). **f** Measurement of time spent in anaphase at 30°C for wild-type control (SHR854), *bgi1Δ* (SHR873), *mad2Δ* (SHR741), and *mad2Δ bgi1Δ* (SHR866) cells. Cells were considered to have entered anaphase if the inter-nuclear distance was >1 µm. Late anaphase was defined as stages when nuclear distances reached a maximum. Mean ± S.D. is indicated. Each dot, *n*, indicates represents a single live-cell measurement. Kruskal-Wallis one-way analysis followed by Dunn's multiple comparison test was used to calculate the statistical significance of differences (*p*-values compared to the wild-type control). Source data are available as a Source Data file.

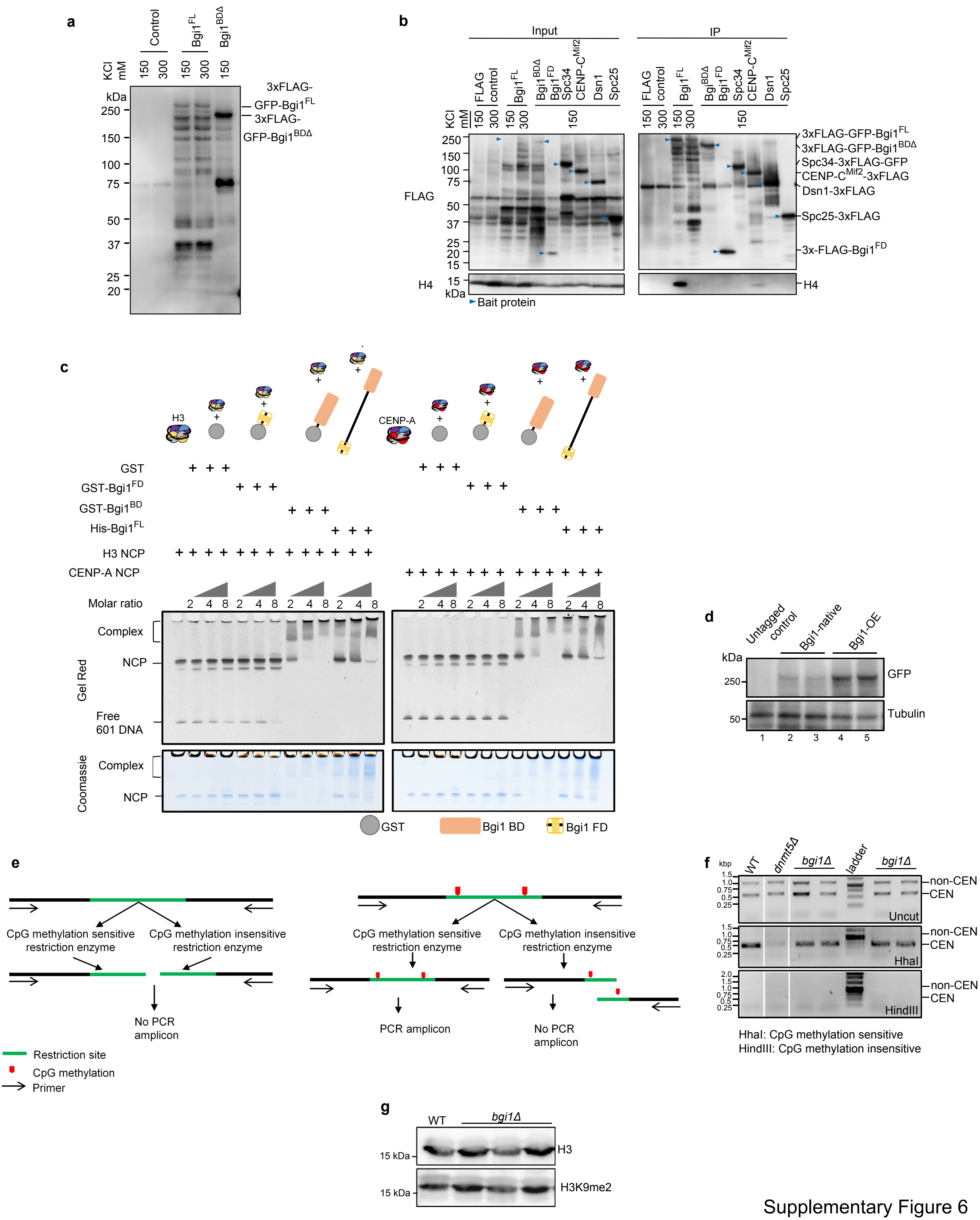

Supplementary Figure 6

Supplementary figure 6. The basic domain of bridgin can interact with nucleosomes, but it does not influence centromere DNA methylation or H3K9me2. a Immunoblot analysis of 3xFLAG tagged bridgin constructs of Bgi1^{FL} expressing SHR879, Bgi1$^{BD\Delta}$ expressing SHR880, and FLAG control expressing SHR942. **b** FLAG immuno-precipitation of kinetochore proteins. Cell lysates from FLAG tagged strains of control SHR942, Bgi1^{FL} expressing SHR879, Bgi1$^{BD\Delta}$ expressing SHR880, Bgi1^{FD} expressing SHR919 (*SHR832::3xFLAG-BGI1*^{FD}), Spc34 (Dam1C) expressing SHR893 (*SPC34::SPC34-3xFLAG*), CENP-C^{Mif2} expressing SHR896, Dsn1 expressing SHR824 and Spc25 expressing SHR861 strains arrested in M phase were extracted, and FLAG affinity purifications were performed with FLAG antibodies. Bait protein bands are indicated. **c** EMSA performed with reconstituted chicken H3 and human CENP-A nucleosome core particles (NCP). Samples were separated on a PAGE gel and stained with Gel Red, followed by Coomassie. **d** The total cellular pool of bridgin, tagged with GFP, expressed under its native promoter in SHR870 (lane 2) and SHR873 (lane 3) or OE promoters of *GAL7* in SHR858 (lane 4) and histone H3 in SHR895 (lane 5) was analyzed by immunoblot analysis. Wild-type (KN99) was used as the untagged control. **e and f** Detection of CpG methylation by Dnmt5 in wild-type (H99) and *bgi1Δ* (SHR876) strains. **(e)** Schematic of the restriction enzyme-PCR based assay used to assess the methylation status at the centromere. **(f)** Estimation of CpG methylation in *bgi1Δ.* The amount of HhaI PCR amplicon is proportional to the level of CpG methylation, in comparison to wild-type. **g** Immunoblot analysis of the whole-cell pool of H3K9me2 levels in wild-type (H99) and *bgi1Δ* (SHR867) cells. Source data are available as a Source Data file.

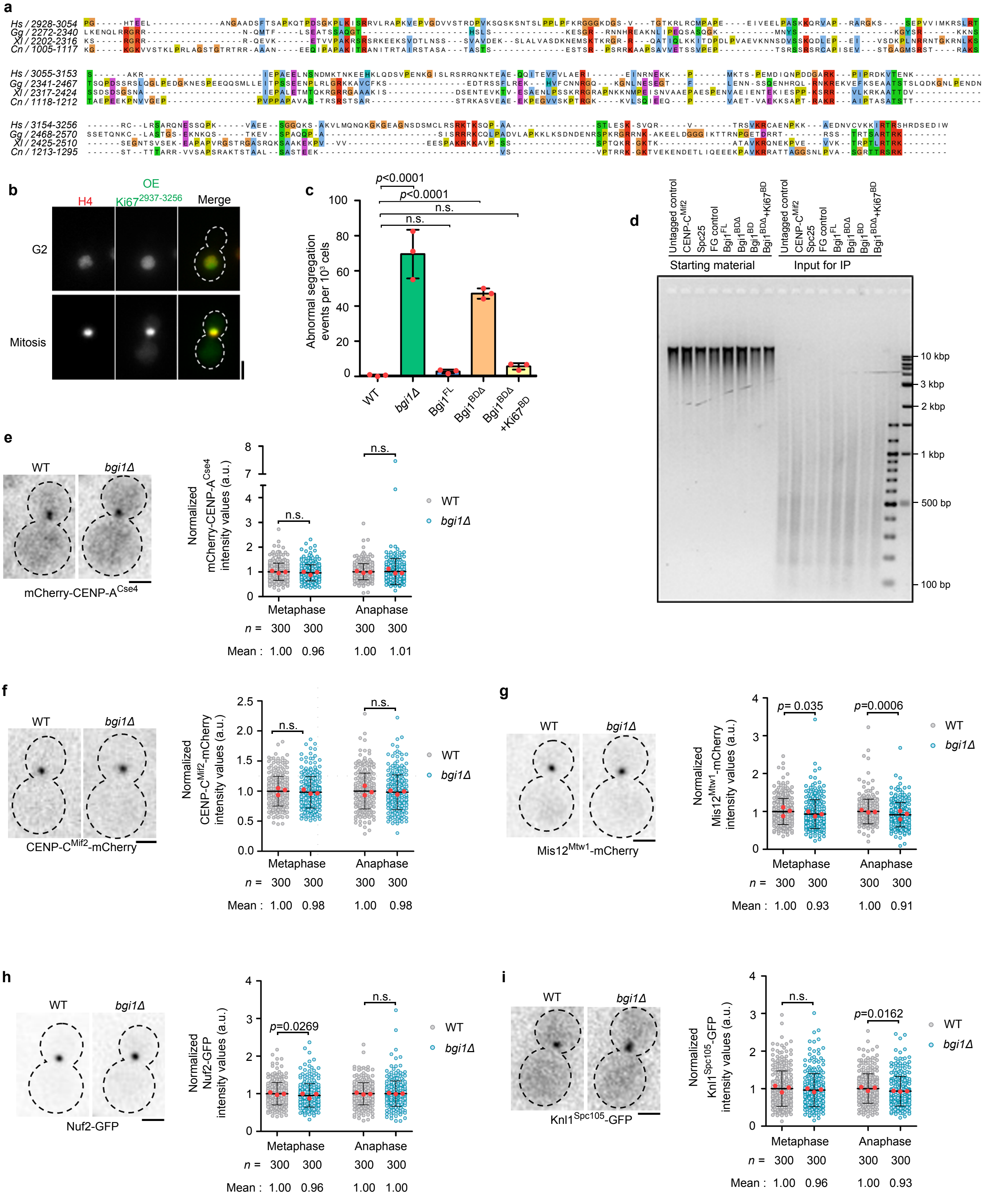

Supplementary Figure 7

Supplementary figure 7. Loss of bridgin leads to reduced proteins levels of the outer kinetochore KMN network. **a** Alignment of the Ki67 basic domain of *Homo sapiens* (*Hs*), *Gallus gallus* (*Gg*), *Xenopus laevis* (*Xl*), and the basic domain of bridgin from *C. neoformans* (*Cn*) was performed using T-Coffee and visualized using Jalview. **b** Co-localization of over-expressed $Ki67^{BD}$ with the nuclear marker histone H4-mCherry (SHR925, *CNVY121::H3p-GFP-KI67*^{BD}) at G2 and M phase. Scale bar, 3 µm. **c** The rate of improper nuclear division was measured at 30˚C using histone H4-mCherry for strains as mentioned in **(Supplementary Fig. 5c)** which include wild-type control (CNVY121), bridgin null-mutant (SHR832), reintegration strains of $Bgi1^{FL}$ (SHR879), $Bgi1^{BD\Delta}$ mutant (SHR880), and $Bgi1^{BD\Delta}$+$Ki67^{BD}$ strain SHR926, and normalized to events per 10^3. The data represents the mean ± S.D. of three independent experiments. One-way ANOVA test with Dunnett's multiple comparisons test was used to calculate the statistical significance of differences (*p*-values compared to wild-type control). **d** Partial digestion of chromatin by MNase prior to co-IP in **(Fig. 7g)**. DNA was isolated from the strains mentioned in **(Fig. 7g)**, which include the 3xFLAG tagged strains of $CENP\text{-}C^{Mif2}$ expressing SHR896, Spc25 expressing SHR861, FLAG-GFP control expressing SHR918, $Bgi1^{FL}$ expressing SHR879, $Bgi1^{BD\Delta}$ expressing SHR880, $Bgi1^{BD}$ expressing SHR917, the domain swap chimera $Bgi1^{BD\Delta}$+$Ki67^{BD}$ expressing SHR926 and untagged wild-type control (H99) prior to and after digestion with MNase. Following which the DNA was separated on an agarose gel and stained with ethidium bromide (EtBr) for visualization. **e-i** Measurement of the kinetochore protein fluorescence intensity of **(e)** $CENP\text{-}A^{Cse4}$ **(f)** $CENP\text{-}C^{Mif2}$ **(g)** $Mis12^{Mtw1}$ **(h)** Nuf2 **(i)** $Knl1^{Spc105}$ in wild-type control and *bgi1Δ* backgrounds at metaphase and anaphase. *Left,* the kinetochore fluorescent signals of representative cells of wild-type and *bgi1Δ* at metaphase are shown as inverted greyscale images. Scale bar, 2 µm. *Right*, signal intensities in wild-type or *bgi1Δ* cells were quantified at metaphase and anaphase and normalized to the mean wild-type signal at either metaphase or anaphase, respectively. Measurements of $CENP\text{-}A^{Cse4}$, $CENP\text{-}C^{Mif2}$, $Mis12^{Mtw1}$, Nuf2 and $Knl1^{Spc105}$ were performed in strains of CNVY120 (*BGI1, KN99::mCherry-CENP-A*^{CSE4}), CNVY102 (*BGI1, CENP-C*^{MIF2}*::CENP-C*^{MIF2}*-mCherry*), SHR772 (*BGI1, MIS12*^{MTW1}*:: MIS12*^{MTW1}*-mCherry, KNL1*^{SPC105}*::KNL1*^{SPC105}*-GFP*), SHR516 (*BGI1, NUF2::NUF2-GFP*) and SHR772, respectively, for wild-type control and SHR902 (*BGI1::bgi1Δ, KN99::mCherry-CENP-A*^{CSE4}) SHR850 (*BGI1::bgi1Δ, CENP-C*^{MIF2}*::CENP-C*^{MIF2}*-mCherry*), SHR904 (*BGI1::bgi1Δ, MIS12*^{MTW1}*:: MIS12*^{MTW1}*-mCherry, KNL1*^{SPC105}*::KNL1*^{SPC105}*-GFP*), SHR903 (*BGI1::bgi1Δ, NUF2::NUF2-GFP*) and SHR904, respectively, for *bgi1Δ*. Results of three independent experiments with 100 cells or kinetochore clusters for metaphase and anaphase, respectively, are represented. The red dot represents the mean of one experiment; mean ± S.D. is shown. For statistical comparison of differences between the samples Mann-Whitney two-tailed analysis was applied, *p*-values show significant differences. Source data are available as a Source Data file.

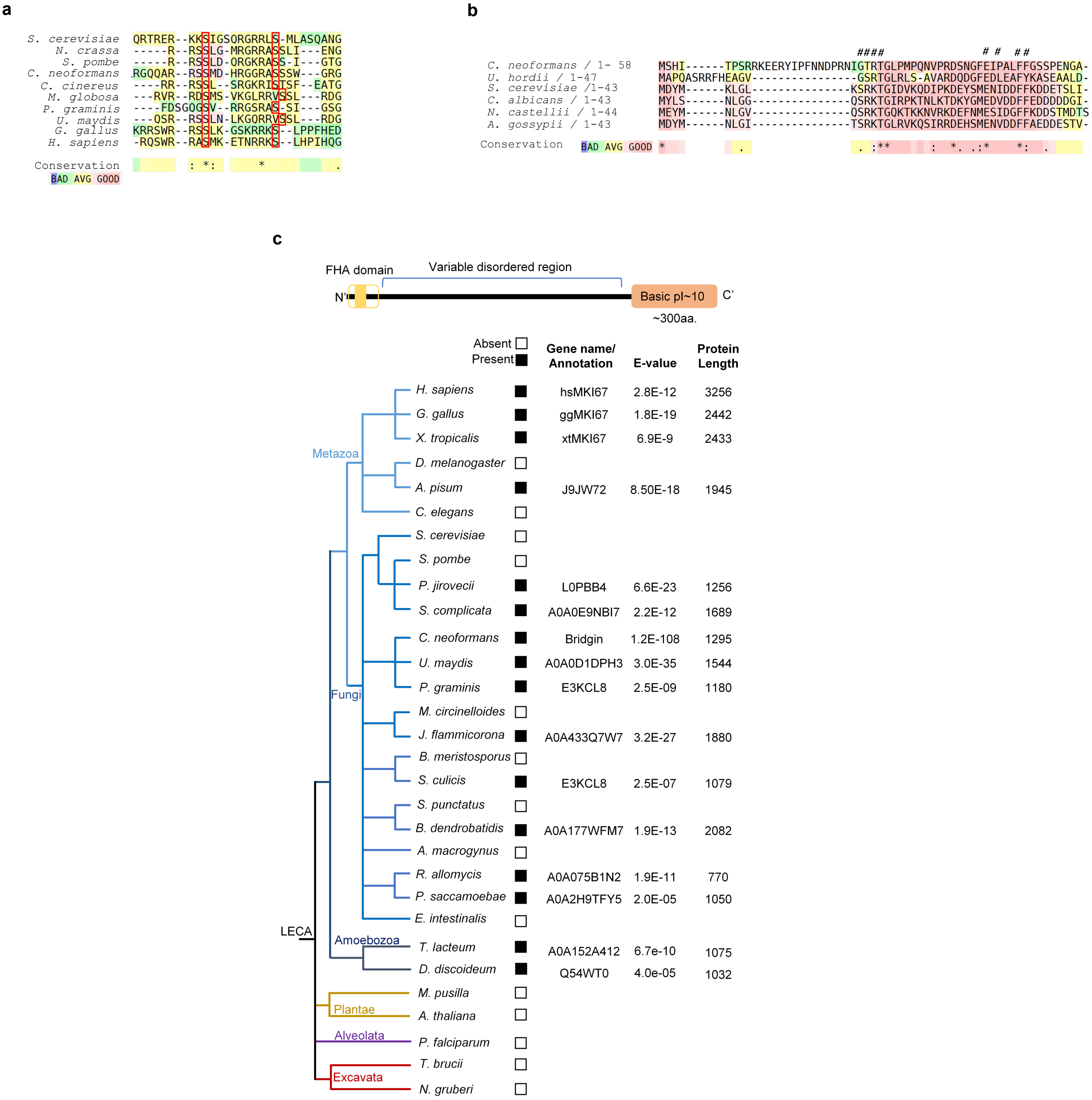

Supplementary Figure 8

Supplementary figure 8. Conservation of motifs and the identification of bridgin homologs across eukaryotes. a Sequence alignment of the Dsn1 basic motif encompassing the two Aurora kinase B/Ipl1 phosphorylation sites across the mentioned species are highlighted in the red box. Alignment and visualization were performed using T-Coffee. **b** Alignment of the described CENP-C^{Mif2}-$Mis12^{Mtw1}$ interacting motif in CENP-C^{Mif2}. # represent CENP-C^{Mif2} residues important for CENP-C^{Mif2}-$Mis12^{Mtw1}$ interaction, as shown in *S. cerevisiae*. **c** Identification of bridgin-like proteins across eukaryotes. The FHA domain of CnBgi1 was used as the initial bait for iterative jackhammer searches. Subsequently, protein features scored for from the obtained hits include the FHA domain at the amino-terminus, followed by a variable-length disordered region and a carboxy terminus of ~300 residues with an isoelectric point of ~10 or greater are considered.

Supplementary table 1:

Start	*p*-value	+	sequence	-
2	1.21E-04	E	EEDEVQ	E
2	1.21E-04	E	EEDEVQ	E
2	1.21E-04	E	EEDEVQ	E
4	2.93E-04	DEY	EEYEVQ	
2	1.15E-03	E	EEEEVQ	E
2	1.15E-03	E	EEEEVQ	E
2	1.95E-03	E	EEKEVD	L
2	2.14E-03	E	EEKESD	D
2	8.67E-03	E	EKEERN	P
3	1.20E-02	TE	EEEEYL	
1	4.36E-02		EEEEEQ	KE
1	4.93E-02		EEEGVE	EL
2	6.16E-02	E	VEEERK	P

Supplementary table 1: List of bridgin repeats. An amino acid repetitive motif was identified within bridgin using MEME suit **(Supplementary Fig. 5a)**. *P*-values were determined using two-tailed rank-sum test.

Supplementary table 2:

Strain name	Genotype	Reference
H99	*MATα (Wild type)*	3
KN99	*MATa (Wild type)*	4
SHR896	*MATα CENP-C*^{MIF2}*::CENP-C*^{MIF2}*-3xFLAG-HygB*	This study
SHR824	*MATα DSN1::DSN1-3xFLAG-HygB, NUF2::NUF2-GFP-NAT, Mis12*^{MTW1}*:: Mis12*^{MTW1}*-mCherry-NEO*	This study
SHR861	*MATα SPC25::SPC25-3xFLAG-HygB*	This study
SHR823	*MATα SPC25::SPC25-3xFLAG-HygB, NUF2::NUF2-GFP-NAT, Mis12*^{MTW1}*::Mis12*^{MTW1}*-mCherry-NEO*	This study
SHR845	*MATa KN99::mCherry-CENP-A-NEO (pLKB74), SOS7::GAL7p-GFP-SOS7-HygB*	This study
SHR843	*MATa H4::H4-mCherry-NEO, Bgi1::Bgi1-V5-GFP-NAT*	This study
SHR876	*MATa CENP-C*^{MIF2}*:: CENP-C*^{MIF2}*-mCherry-NEO, BGI1::BGI1-V5-GFP-NAT*	This study
SHR897	*MATa CENP-C*^{MIF2}*:: CENP-C*^{MIF2}*-mCherry-NEO, BKT2::BKT2-V5-GFP-NAT*	This study
SHR842	*MATa CENP-C*^{MIF2}*:: CENP-C*^{MIF2}*-mCherry-NEO, YTA7::YTA7-V5-GFP-NAT*	This study
SHR905	*MATa KN99::mCherry- CENP-A*^{CSE4}*-NEO (pLKB74), MCM6::MCM6-V5-GFP-NAT*	This study
SHR870	*MATα BGI1::BGI1-V5-GFP-NAT*	This study
SHR516	*MATa Mis12*^{MTW1}*:: Mis12*^{MTW1}*-mCherry-NEO, NUF2::NUF2-GFP-NAT*	1
CNVY120	*MATa KN99::GFP-DAD1-NAT (pVY2), KN99:: CENP-A*^{CSE4}*-mCherry-NEO*	1
SHR772	*MATa Mis12*^{MTW1}*:: Mis12*^{MTW1}*-mCherry-NEO, KNL1*^{SPC105}*:: KNL1*^{SPC105}*-GFP-NAT*	This study
SHR869	*MATa Mis12*^{MTW1}*:: Mis12*^{MTW1}*-mCherry-NEO, BGI1::BGI1-V5-GFP-NAT*	This study
SHR906	*MATα DAD2::GAL7p-mCherry-DAD2-HygB, BGI1::BGI1-V5-GFP-NAT*	This study
SHR907	*MATα Mis12*^{MTW1}*::GAL7p-mCherry- Mis12*^{MTW1}*-HygB, BGI1::BGI1-V5-GFP-NAT*	This study
SHR908	*MATα BGI1::BGI1-V5-GFP-NAT, sos7Δ::NEO*	This study
SHR909	*MATa Dad2::DAD2-mCherry-NEO, BGI1::BGI1-V5-GFP-NAT*	This study
SHR720	*MATa NDC80::NDC80-mCherry-NEO, CENP-C*^{MIF2}*::GAL7p-GFP- CENP-C*^{MIF2}*-HygB*	This study
SHR724	*MATa NDC80::NDC80-mCherry-NEO, Mis12*^{MTW1}*::GAL7p-GFP-Mis12*^{MTW1}*-HygB*	This study

SHR732	*MATa Mis12*^{MTW1}*:: Mis12*^{MTW1}*-mCherry-NEO, NUF2::GAL7p-GFP-NUF2-HygB*	This study
SHR910	*MATα KNL1*^{SPC105}*:: KNL1*^{SPC105}*-GFP-NAT, NUF2::GAL7p-mCherry-NUF2-HygB*	This study
SHR768	*MATα KNL1*^{SPC105}*:: KNL1*^{SPC105}*-GFP-NAT, Mis12*^{MTW1}*::GAL7p-mCherry-Mis12*^{MTW1}*-HygB*	This study
SHR771	*MATa MIS12*^{MTW1}*:: MIS12*^{MTW1}*-mCherry-NEO, KNL1*^{Spc105}*::GAL7p-GFP-KNL1*^{Spc105}*-HygB*	This study
SHR911	*MATa DAD2::DAD2-mCherry-NEO, NUF2::GAL7p-GFP-NUF2-HygB*	This study
SHR788	*MATa Dad2::DAD2-mCherry-NEO, KNL1*^{SPC105}*::GAL7p-GFP- KNL1*^{SPC105}*-HygB*	This study
SHR713	*MATa NDC80::NDC80-mCherry-NEO, DAD1::GAL7p-GFP-DAD1*	This study
SHR867	*MATα bgi1Δ::NEO*	This study
SHR832	*MAT a H4::H4-mCherry-NEO, bgi1Δ::HygB*	This study
SHR873	*MATa H4::H4-mCherry-NEO, KN99::GFP-PCNA-NAT (pSS60), bgi1Δ::HygB*	This study
SHR879	*MATa H4::H4-mCherry-NEO, bgi1Δ::HygB, SHR832::3xFLAG-GFP-BGI1-NAT (pSS62)*	This study
SHR854	*MATa H4::H4-mCherry-NEO, KN99::GFP-PCNA-NAT (pSS60)*	This study
SHR866	*MATα mad2Δ::NEO, bgi1Δ::HygB, H4::H4-GFP-NAT*	This study
SHR741	*MATα H4::H4-GFP-NAT, mad2Δ::NEO*	This study
SHR913	*MAT a H4::H4-mCherry-NEO, bgi1Δ::HygB, SHR832::3x-FLAG-GFP-BGI1 FDΔ-NAT (pSS63)*	This study
SHR880	*MAT a H4::H4-mCherry-NEO, bgi1Δ::HygB, SHR832::3x-FLAG-GFP-BGI1 BDΔ-NAT (pSS64)*	This study
SHR915	*MAT a H4::H4-mCherry-NEO, bgi1Δ::HygB, SHR832::3x-FLAG-GFP-BGI1 FD-NAT (pSS65)*	This study
SHR916	*MAT a H4::H4-mCherry-NEO, bgi1Δ::HygB, SHR832::3x-FLAG-GFP-BGI1 USD-NAT (pSS66)*	This study
SHR917	*MAT a H4::H4-mCherry-NEO, bgi1Δ::HygB, SHR832::3x-FLAG-GFP-BGI1 BD-NAT (pSS67)*	This study
SHR918	*MAT a H4::H4-mCherry-NEO, bgi1Δ::HygB, SHR832::3x-FLAG-GFP-NAT (pSS61)*	This study
SHR942	*MATα bgi1Δ::HygB, bgi1Δ::3x-FLAG-NAT (pSS59)*	This study
CNVY121	*MATa H4::H4-mCherry-NEO*	1
SHR903	*MATa Mis12*^{MTW1}*:: Mis12*^{MTW1}*-mCherry-NEO, NUF2::NUF2-GFP-NAT, bgi1Δ::HygB*	This study
SHR798	*MATa NDC80::NDC80-mCherry-NEO, KNL1*^{SPC105}*::GAL7p-GFP-KNL1*^{SPC105}*-HygB*	This study

SHR904	*MATa Mis12^{MTW1}:: Mis12^{MTW1}-mCherry-NEO, KNL1^{SPC105}:: KNL1^{SPC105}-GFP-NAT, bgi1Δ::HygB*	This study
SHR902	*MATa KN99::GFP-DAD1-NAT (pVY2), KN99::mCherry-CENP-A^{CSE4}-NEO, bgi1Δ::HygB*	This study
SHR919	*MAT a H4::H4-mCherry-NEO, bgi1Δ::HygB, SHR832::3x-FLAG-FD-NAT (pSS77)*	This study
SHR847	*MATa NDC80::NDC80-mCherry-NEO, BGI1::BGI1-V5-GFP-NAT*	This study
SHR858	*MATa NDC80::NDC80-mCherry-NEO, BGI1::GAL7p-GFP-BGI1-HygB*	This study
SHR893	*MATα SPC34::SPC34-3xFLAG-GFP-NAT*	This study
SHR895	*MAT a H4::H4-mCherry-NEO, bgi1Δ::HygB, SHR832::H3p-GFP-BGI1-NAT (pSS68)*	This study
SHR920	*MAT a H4::H4-mCherry-NEO, bgi1Δ::HygB, SHR832::H3p-GFP-BGI1-FDΔ-NAT (pSS69)*	This study
SHR921	*MAT a H4::H4-mCherry-NEO, bgi1Δ::HygB, SHR832::H3p-GFP-BGI1-BDΔ-NAT (pSS70)*	This study
SHR922	*MAT a H4::H4-mCherry-NEO, bgi1Δ::HygB, SHR832::H3p-GFP-BGI1-FD-NAT (pSS71)*	This study
SHR923	*MAT a H4::H4-mCherry-NEO, bgi1Δ::HygB, SHR832::H3p-GFP-BGI1-USD-NAT (pSS72)*	This study
SHR924	*MAT a H4::H4-mCherry-NEO, bgi1Δ::HygB, SHR832::H3p-GFP-BGI1-BD-NAT (pSS73)*	This study
SHR925	*MATa H4::H4-mCherry-NEO, KN99::H3p-GFP-HsKi67^{BD}-NAT (pSS74)*	This study
SHR926	*MATa H4::H4-mCherry-NEO, bgi1Δ::HygB, SHR832::3x-FLAG-GFP-Bgi1BDΔ+HsKi67BD-NAT (pSS75)*	This study
SHR945	*MATα KNL1^{SPC105}:: GAL7p-mCherry-KNL1^{SPC105}-HygB, BGI1::BGI1-V5-GFP-NAT*	This study
CNVY108	*MATα H99::GFP-H4-NAT (pLKB35)*	1
SHR835	*MATα H99::GFP-H4-NAT (pLKB35), sos7Δ::NEO*	This study
SHR702	*MATa CENP-A^{Cse4}::GAL7pr-mCherry-CENP-A ^{Cse4}-HygB*	This study
SHR716	*MATα CENP-C^{Mif2}::GAL7p-GFP-CENP-C ^{Mif2}-HygB*	This study
SHR736	*MATa MIS12^{Mtw1}::GAL7p-mCherry-MIS12^{Mtw1}-HygB*	This study
SHR718	*MATα NUF2::GAL7p-GFP-NUF2-HygB*	This study
SHR807	*MATα KNL1^{SPC105}::GAL7p-GFP- KNL1^{SPC105}-HygB*	This study
SHR710	*MATα DAD1::GAL7p-GFP-DAD1::HygB*	This study
SHR738	*MATa DAD2::GAL7p-mCherry-DAD2-HygB*	This study
CNVY102	*MATa CENP-C^{MIF2}:: CENP-C^{MIF2}-mCherry-NEO*	1
SHR850	*MATa CENP-C^{MIF2}:: CENP-C^{MIF2}-mCherry-NEO, bgi1Δ::HygB*	This study

Supplementary table 2: List of strains used in this study.

Supplementary table 3:

Plasmid name	Description	Reference
pCIN19	*H3p-GFP-NAT*	Alspaugh lab
pSS61	*BGI1p-3xFLAG-GFP-NAT in pBlueScriptII KS(-)*	This study
pSS55	*CENP-C*^{MIF2}*-3xFLAG-HygB in pBlueScriptII KS(-)*	This study
pSS56	*DSN1-3xFLAG-HygB in pBlueScriptII KS(-)*	This study
pSS57	*SPC25-3xFLAG-HygB in pBlueScriptII KS(-)*	This study
pSS78	*BGI1-V5-GFP-NAT in pRS426*	This study
pSS85	*GAL7p-GFP-SOS7-HygB in pBlueScriptII KS(-)*	This study
pSS79	*BKT2-V5-GFP-NAT in pRS426*	This study
pSS81	*BKT3-V5-GFP-NAT in pRS426*	This study
pSS80	*YTA7-V5-GFP-NAT in pRS426*	This study
pSS82	*MCM6-V5-GFP-NAT in pRS426*	This study
pSS13	*GAL7p-GFP- CENP-C*^{MIF2}*-HygB pBlueScriptII KS(-)*	This study
pSS14	*GAL7p-GFP- MIS12*^{MTW1}*-HygB pBlueScriptII KS(-)*	This study
pSS7	*GAL7p-GFP-NUF2-HygB pBlueScriptII KS(-)*	This study
pSS24	*GAL7p-mCherry-NUF2-HygB pBlueScriptII KS(-)*	This study
pSS19	*GAL7p-mCherry- MIS12*^{MTW1}*-HygB pBlueScriptII KS(-)*	This study
pSS27	*GAL7p-GFP-KNL1*^{SPC105}*-HygB pBlueScriptII KS(-)*	This study
pSS21	*GAL7p-mCherry-DAD2-HygB pBlueScriptII KS(-)*	This study
pSS4	*GAL7p-GFP-DAD1-HygB pBlueScriptII KS(-)*	This study
pSS62	*BGI1 FL in pSS61 (BamHI/SpeI)*	This study
pSS63	*BGI1 FDΔ in pSS61 (BamHI/SpeI)*	This study
pSS64	*BGI1 BDΔ in pSS61 (BamHI/SpeI)*	This study
pSS65	*BGI1 FD in pSS61 (BamHI/SpeI)*	This study
pSS66	*BGI1 USD in pSS61 (BamHI/SpeI)*	This study
pSS67	*BGI1 BD in pSS61 (BamHI/SpeI)*	This study
pSS58	*SPC34-3xFLAG-HygB in pBlueScriptII KS(-)*	This study
pSS86	*BGI1 FD in pGEX6P1*	This study
pSS87	*BGI1 BD in pGEX6P1*	This study
pSS89	*6xHis-BGI1 in pFASTBacHTA*	This study
pSS87	*GAL7p-GFP-BGI1-HygB in pBlueScriptII KS(-)*	This study
pSS68	*BGI1 FL (BamHI/SpeI) in pCIN19*	This study
pSS69	*BGI1 FDΔ (BamHI/SpeI) in pCIN19*	This study
pSS70	*BGI1 BDΔ (BamHI/SpeI) in pCIN19*	This study
pSS71	*BGI1 FD (BamHI/SpeI) in pCIN19*	This study
pSS72	*BGI1 USD (BamHI/SpeI) in pCIN19*	This study
pSS73	*BGI1 BD (BamHI/SpeI) in pCIN19*	This study
pSS74	*BGI1 HsKi67 BD (BamHI/SpeI) in pCIN19*	This study

pSS75	*BGI1 BDΔ+HsKi67BD in pSS61 (BamHI/SpeI)*	This study
pSS128	*GAL7p-mCherry-KNL1-HygB pBlueScriptII KS(-)*	This study

Supplementary table 3: List of plasmids used in this study.

Supplementary table 4:

Name	Sequence (5’------3’)	Description
SHR3	GTGCGAGCTCGCTAGCTTCTCCAAGATGGGTGTCACG	Generation of GAL7p-GFP-DAD1
SHR4	GTGAGAATGCGGCCGCCTTGGAGTGCTAGTTTTCCTGC	
SHR5	ATGTCTTTATCAAGACCATCGAATGCCTACGATGC	
SHR6	GTGCGGTACCGAGCTCATGCCTATGAAGTCCAGC	
SHR72	AGCTTGAGCTCCTTCGAGATATACAGCTCC	Generation of GAL7p-GFP/mCherry-DAD2
SHR73	TTTAAGCGGCCGCCACTCGAGAGTTACAGTG	
SHR74	CATCAAGCTTGGTGGTATGTCCCGTCCATCAATAGAAATG	
SHR75	AACTCTCGAGGTGAGATAGGGTTGAAGGAGC	
SHR64	AGCTGAGCTCCAAATCCACAACATCTGAAATACG	Generation of GAL7p-GFP/mCherry-Mis12^{MTW1}
SHR65	AAATTTGCGGCCGCGAACGTAGAGACGATTATGAATGC	
SHR66	GCTGTTAACGGTGGTATGGTCCCGAGGAAGCCAG	
SHR67	AGGTCCTCGAGCATTGGCAAGCTAACTAAATTAATGGAACG	
SHR68	AGCTGAGCTCCAAGTCTCTTGTCGACATCTCTCC	Generation of GAL7p-GFP/mCherry-CENP-C^{MIF2}
SHR69	TTATTAGCGGCCGCGTTGAAGATGTTCTGGAGAAGTGC	
SHR70	AACCCAAGCTTATGTCCCACATAACACCCTCAAGA	
SHR71	TCGTCTCGAGCTTTCCATCTGCTTGCTTCTTTGG	
SHR50	GACTGAGCTCCTTGCACTCTTACAGAAGCCTCC	Generation of GAL7p-GFP/mCherry-NUF2
SHR51	TCACATGCGGCCGCGATTGCTGAATGCAAATGCAG	
SHR52	GACTAAGCTTATGTCGCAGCAGAATCGCAG	
SHR53	GACTGGTACCGATTTCAAGCTGTGTGACGATACG	
SHR112	AATGCGAGCTCTCTGTACCAGATAGTCACCAC	Generation of GAL7p-GFP/mCherry-KNL1^{SPC105}
SHR113	ATATATATGCGGCCGC AATATGCTCGGTTAACTGCTG	
SHR114	TAGTCAAGCTTATGTCTTTAGCAGCTCGCTC	
SHR115	TCTAGGTACCGTTTCGAGTTGCTGTAGCTG	
SHR179	CTACTCTTACAGGCAAGTTGGAG	KNL1^{SPC105}-GFP tagging
SHR180	CTCGCCCTTGCTCACCATACTGGAGTACCTTGCACCGA	
SHR181	TCGGTGCAAGGTACTCCAGTATGGTGAGCAAGGGCGAG	
SHR182	CCTTGTAACCATCCATACAACCTAGGATGTGAGCTGGAGAGC	
SHR183	GCTCTCCAGCTCACATCCTAGGTTGTATGGATGGTTACAAGG	
SHR184	CTCTGGTGATACACTCAAGGAC	
SHR77	TCGTGAGCTCGTCTCAACAATTTGGTTACTGATCAAGG	Generation of mad2Δ cassette
SHR78	AGGACACTAGTTTCGTGGGGTAGAAACTGGAAG	
SHR81	TTTAAAGCGGCCGCGTAATATTATCTAGTTCAACGTTCACG	
SHR80	ACCCTTAGATCTGTGAATTCCTTTTATCCATTTTCC	
SHR389	GTCAGAGCTCCTCACAACATAAGACATCG	

SHR390	ATATATGCGGCCGCAGATCCAATATTACTACTATACGG	Generation of GAL7p-GFP-SOS7
SHR391	GTCAAAGCTTGCTGGTGCAGGAATGGAACCCTCTATGACG	
SHR392	GTCACTCGAGGTTTGAGCTTCAACCAG	
SHR374	GATGTTGAGAGAAGTGATGGAGG	Tagging of DSN1 with 3xFLAG
SHR450	CTACTTGTCATCGTCATCCTTGTAGTCGATGTCATGATCTTTATAATCACCGTCATGGTCTTTGTAGTCTTCCCTCTCCGGCCTA	
SHR452	CGACTACAAGGATGACGATGACAAGTAGCTAGTAACGGCCGCCA	
SHR377	CAATTGTAACCATCGTCATTAACACCAGTGTGATGGATATCTGCAGA	
SHR378	TCTGCAGATATCCATCACACTGGTGTTAATGACGATGGTTACAATTG	
SHR379	GATGGCATTTCGCTAACCAC	
SHR367	GTATGTGTCGACGTATGACCT	
SHR451	CTACTTGTCATCGTCATCCTTGTAGTCGATGTCATGATCTTTATAATCACCGTCATGGTCTTTGTAGTCTTTACCCAAAGCCAATTG	Tagging of SPC25 with 3xFLAG
SHR370	GCACTCAAAAATGTTACAAATACAGTCCAGTGTGATGGATATCTGCAGA	
SHR371	TCTGCAGATATCCATCACACTGGACTGTATTTGTAACATTTTTGAGTGC	
SHR372	CATCGTCATGCCAATCGTG	
SHR493	GAAGAATGGTAGAGCAAGG	Tagging of CENP-C^{MIF2} with 3xFLAG
SHR511	CTTGTCATCGTCATCCTTGTAGTCGATGTCATGATCTTTATAATCACCGTCATGGTCTTTGTAGTCTCCAGCACCTCTCCTACTCTTCCCCTTAC	
SHR494	ACCCATTCATACCTTCTTTCTCAGTGTGATGGATATCTGCAGA	
SHR495	TCTGCAGATATCCATCACACTGAGAAAGAAGGTATGAATGGGT	
SHR496	CACCAGATAGAAAGAGTCTAGG	
SHR513	CGACGGTATCGATAAGCTTGATATCGAGATGTACGAGGAAGAAGAGG	Tagging of BGI1 with V5-GFP
SHR514	GAGACCAAGGAGAGGGTTGGGGATAGGCTTACCAGCACCCTTCCTACTCCTGGTTGTCCT	
SHR515	GCACCTATCTTACAACATCCACTATCAGGATGTGAGCTGGAGAGC	
SHR516	GCTCTCCAGCTCACATCCTGATAGTGGATGTTGTAAGATAGGTGC	
SHR517	CGGCCGCTCTAGAACTAGTCAGAGGAAGGAACCTTGGATG	
SHR518	CGACGGTATCGATAAGCTTGATATCCAATGGAGCTCTCCAGATGTC	Tagging of YTA7 with V5-GFP

SHR519	GAGACCAAGGAGAGGGTTGGGGATAGGCTTACCAGCACCATC GTTTTTCCAACTATTAACCTCTTTG	
SHR520	AAACGCCATGCTAACAACAAAATGAGGATGTGAGCTGGAGAGC	
SHR521	GCTCTCCAGCTCACATCCTCATTTTGTTGTTAGCATGGCGTTT	
SHR522	CGGCCGCTCTAGAACTAGTCTCCATCTTCGTTCATTCACGC	
SHR523	CGACGGTATCGATAAGCTTGATATCCAGTCAGGGAAGATTTGA CGTG	Tagging of BKT2 with V5-GFP
SHR524	GAGACCAAGGAGAGGGTTGGGGATAGGCTTACCAGCACCCTC GTCACCACCGAACAC	
SHR525	GCATTAGTGTGGCTTCTTGATTCAGGATGTGAGCTGGAGAGC	
SHR526	GCTCTCCAGCTCACATCCTGAATCAAGAAGCCACACTAATGC	
SHR527	CGGCCGCTCTAGAACTAGTCATTCAAGGTAGCACATAAAGTTG AC	
SHR533	CGACGGTATCGATAAGCTTGATATCACTGCTGAGAGGAGCTGT G	Tagging of BKT3 with V5-GFP
SHR534	GAGACCAAGGAGAGGGTTGGGGATAGGCTTACCAGCACCGAT CATTTCGTAACCTTCATCTTTTGC	
SHR535	CGCTATACTACCTTAAGTTTAACCGTAGGATGTGAGCTGGAGA GC	
SHR536	GCTCTCCAGCTCACATCCTACGGTTAAACTTAAGGTAGTATAGC G	
SHR537	CGGCCGCTCTAGAACTAGTCCAACACACAAATTATCAAGGATTC C	
SHR453	CGACGGTATCGATAAGCTTGATATCGATCTTCGTCAGCATCTAG CTC	Tagging of SPC34 with 3xFLAG-GFP
SHR454	TATAATCACCGTCATGGTCTTTGTAGTCAGCTCCATCTGCAAAT CTAACCCTACCC	
SHR455	GGTATACAGTTAGATCAAGGAGGATACAGGATGTGAGCTGGAG AGC	
SHR456	GCTCTCCAGCTCACATCCTGTATCCTCCTTGATCTAACTGTATA CC	
SHR457	CGGCCGCTCTAGAACTAGTCAAATAACATGACGTGACGGAC	
SHR538	CGACGGTATCGATAAGCTTGATATCGCTCCAGAGGTATATTCGA TACG	Tagging of MCM6 with V5-GFP
SHR539	GAGACCAAGGAGAGGGTTGGGGATAGGCTTACCAGCACCTGC GGGAATAGAAGAAGATAAATCTG	
SHR540	GGAACAGCGGGAAATGCAAGGATGTGAGCTGGAGAGC	
SHR541	GCTCTCCAGCTCACATCCTTGCATTTCCCGCTGTTCC	
SHR542	CGGCCGCTCTAGAACTAGTCGAACCCTGCTCAAGTCG	

SHR560	GGTAAGCCTATCCCCAACCCTCTCCTTGGTCTCGACAGCACCG GTGCTATGGTGAGCAAGGGCGAG	Common V5-GFP primer
SHR548	GCTCAGAGGTCACATACAGG	Generation of sos7Δ cassette
SHR564	CTGCAGATATCCATCACACTGGAGGTCAAAGATGGGTAAATAG C	
SHR565	GCTATTTACCCATCTTTGACCTCCAGTGTGATGGATATCTGCAG	
SHR551	GCTGTCCACTTTCGAAGGTCAGTGTGCTGGAATTCGC	
SHR552	GCGAATTCCAGCACACTGACCTTCGAAAGTGGACAGC	
SHR553	CATTATTGGAGATGTCTGAAGCG	
SHR582	CGACGGTATCGATAAGCTTGATATCCCAGAAGGATAGAGTCCT CTG	Generation of GAL7p-GFP/mCherry-BGI1
SHR583	CTCACATCCTCGCAGCTTTTCGTTGCAAGTCAGC	
SHR584	GCTGACTTGCAACGAAAAGCTGCGAGGATGTGAG	
SHR585	CTCTCGTCAAACTCTTGCATGGCACCAGCGTACAGCTCGTCCA TGCCG	
SHR586	CGGCATGGACGAGCTGTACGCTGGTGCCATGCAAGAGTTTGAC GAGAG	
SHR587	CGGCCGCTCTAGAACTAGTGTTACTGTCAATTGAGGAAGC	
SHR600	CTAGCTTGGCAATAGTGTAGCAG	Generation of *bgi1Δ* cassette
SHR601	CTGCAGATATCCATCACACTGGGTTGCTGTTTGTATAGCGAGTC	
SHR602	GACTCGCTATACAAACAGCAACCCAGTGTGATGGATATCTGCA G	
SHR603	GCACCTATCTTACAACATCCACTATCCAGTGTGCTGGAATTCGC	
SHR604	GCGAATTCCAGCACACTGGATAGTGGATGTTGTAAGATAGGTG C	
SHR605	CAGAGGAAGGAACCTTGGATG	
SHR611	AGTCGGATCCGCCGCTGGTGCCATGCAAGAGTTTGACGAGAG	Generation of domain deletion constructs of BGI1 expressed under native or H3 promoter
SHR612	ATATATACTAGTCAAGTACTCGCCACTTATCACTC	
SHR613	AGTCGGATCCGCCGCTGGTGCCTGGACCTATGGAAGATGCT	
SHR614	ATATATACTAGTCTATCTTACAACATCCACTATCCATTATCATTA AGCATCTTCCATAGGTCCA	
SHR615	ATATATACTAGTCTATCTTACAACATCCACTATCCATTATCATTAT AGTTGAACCTGAAAAGCTTTTTC	
SHR616	AGTCGGATCCGCCGCTGGTGCCGAGATCGAAGAGAAGGGTAA AG	
SHR715	CCTCAGTGTGGCCTGGGGCACCAGCCTCATCTTGCTCCTGCAC	Generation of fusion protein of
SHR716	GTGCAGGAGCAAGATGAGGCTGGTGCCCCAGGCCACACTGAG G	

SHR676	ATATATACTAGTCTATCTTACAACATCCACTATCCATTATCATTACCAAATATCTTCACTGTCCCTATG	BGI1-BDΔ and HsKi67-BD
SHR677	AGTCGGATCCCCAGGCCACACTGAGG	Over-expression of HsKi67BD
SHR729	CTGAGGATCCATGCAAGAGTTTGACGAGAG	Tagging BGI1-FD with GST for recombinant protein purification
SHR730	CTGAGTCGACTTAATCTTCCATAGGTCCATAGTTG	
SHR731	CTGAGGATCCATCGAAGAGAAGGGTAAAGG	Tagging BGI1-BD with GST for recombinant protein purification
SHR733	ATATATGCGGCCGCTTACTTCCTACTCCTGGTTGTC	
VYP75	AGTCTCGTGTGGCTATGATT	Centromeric DNA methylation
VYP76	GGATCTGCTTGACAGTGTCA	
VYP79	CCAACCGAAGCCCAAGACAA	Non-centromeric DNA methylation
VYP80	TTGAAGGATGATCCGGCCGA	
VYP65	CCATCCAGTTCTTGCTTGAG	ChIP primers for quantitative PCR
VYP66	GCAAGGAATGTGTTGTCTGG	
VYP67	CAGACCCTTCCTTCAGCCG	
VYP68	TGGCAAGGAGTCGTCAGCG	
NC1	GATCAAGTATAGGCGAAGG	
NC2	ATCTCTTATTCCCACTTCTACTC	
GFP-1	CTGAAGTTCATCTGCACCAC	GFP confirmation
GFP-2	GTCGTCCTTGAAGAAGATGG	
mCherry-1	GTAATGCAGAAGAAGACCATG	mCherry confirmation
mCherry-2	AACTGAGGGGACAGGATGTC	
NAT-1	ACGAATCGGACGACGAATC	NAT confirmation
NAT-2	AGTACGAGACGACCACGAAG	
NEO-1	CTTGTATGGAGCAGCAGACG	NEO confirmation
NEO-2	CTGAAAGCACGAGATTCTTCG	
HYG-1	CCAAGCTCTTCAGCAATATCAC	HYG confirmation
HYG-2	CTTCAGTGACAACGTCGAGC	
GAL7-1	GTATCCATTGCATATCTTATCG	Gal7 confirmation
GAL7-2	GAAACTGCAAGAGATTGTCAG	

SHR457	TGATGCGTATACACAAGC	Spc25 confirmation
SHR458	GAGATCATCAGCAATACG	Dsn1 confirmation
SHR606	CAGCTGGCCGCAGG	Bgi1 confirmation
SHR607	CCTGCGGCCAGCTG	
SHR619	TGTCAAGAGCTGGTTTGTCG	
SHR627	TTCCCTACCAAGGTCAGC	Bkt2 confirmation
SHR634	CATACCATTTTGTATACCATGGC	Yta7 confirmation
	GTCATAGCTGTTTCCTG	M13 sequencing primers
	GTAAAACGACGGCCAGT	

Supplementary table 4: List of primers used in this study.

Supplementary table 5:

Primary antibodies				
Antibody	Species	Source	Catalogue no	Assay and dilution
α-histone H3K9me2	Mouse	Abcam	ab1220	Immunoblot, 1:2000
α-PSTAIR	Mouse	Abcam	10345	Immunoblot, 1:5000
α-GFP	Mouse	Roche	11814460001	Immunoblot, 1:3000
α-histone H4	Mouse	Reference 5	CMA400	Immunoblot, 1:5000
α-FLAG M2	Mouse	Sigma-Aldrich	F3165	Immunoblot, 1:5000
α-pan histone H3	Rat	Reference 6	140-1G1	Immunoblot, 1:3000
α-alpha-Tubulin	Mouse	Sigma-Aldrich	T9026	Immunoblot, 1:5000
Secondary antibodies				
α-Mouse	Goat	Bangalore genei	HO06	Immunoblot, 1:10000
HRP-conjugated α-mouse IgG	Rabbit	Jackson ImmunoResearch	315-035-003	Immunoblot, 1:15000
HRP-conjugated α-Rat IgG	Goat	Jackson ImmunoResearch	112-035-003	Immunoblot,

Supplementary table 5: List of antibodies used in this study.

Supplementary references

1. Kozubowski, L. *et al.* Ordered kinetochore assembly in the human-pathogenic basidiomycetous yeast Cryptococcus neoformans. *MBio* **4**, (2013).

2. Varshney, N. *et al.* Spatio-temporal regulation of nuclear division by Aurora B kinase Ipl1 in Cryptococcus neoformans. *PLoS Genet.* **15**, 1007959 (2019).

3. Toffaletti, D. L., Rude, T. H., Johnston, S. A., Durack, D. T. & Perfect, J. R. Gene transfer in Cryptococcus neoformans by use of biolistic delivery of DNA. *J. Bacteriol.* **175**, 1405–1411 (1993).

4. Nielsen, K. *et al.* Sexual cycle of Cryptococcus neoformans var. grubii and Virulence of congenic a and α isolates. *Infect. Immun.* **71**, 4831–4841 (2003).

5. Hayashi-Takanaka, Y. *et al.* Distribution of histone H4 modifications as revealed by a panel of specific monoclonal antibodies. *Chromosom. Res.* **23**, 753–766 (2015).

6. Kimura, H., Hayashi-Takanaka, Y., Goto, Y., Takizawa, N. & Nozaki, N. The Organization of Histone H3 Modifications as Revealed by a Panel of Specific Monoclonal Antibodies. *Cell Struct. Funct.* **33**, 61–73 (2008).
