# Supplementary material for: Bridgin connects the outer kinetochore to centromeric chromatin
Source: Nat Commun. 2021 Jan 8;12:146. doi: 10.1038/s41467-020-20161-9 (PMC7794384; doi:10.1038/s41467-020-20161-9)
Supplement: Supplementary file 3 — Description of Additional Supplementary Files [file 41467_2020_20161_MOESM3_ESM.pdf]

Description of Additional Supplementary Files

File Name: Supplementary Data 1
Description: Kinetochore homologs

File Name: Supplementary Data 2
Description: Kinetochore IP-MS

File Name: Supplementary Data 3
Description: Bridgin IP-MS

File Name: Supplementary Data 4
Description: Bridgin homologs across eukaryotes
