# Supplementary material for: Bridgin connects the outer kinetochore to centromeric chromatin
Source: Nat Commun. 2021 Jan 8;12:146. doi: 10.1038/s41467-020-20161-9 (PMC7794384; doi:10.1038/s41467-020-20161-9)
Supplement: Supplementary file 8 — Reporting Summary [file 41467_2020_20161_MOESM8_ESM.pdf]

nature research

Corresponding author(s): Kaustuv Sanyal, Tatsuo Fukagawa

Last updated by author(s): Oct 30, 2020

Reporting Summary

Nature Research wishes to improve the reproducibility of the work that we publish. This form provides structure for consistency and transparency in reporting. For further information on Nature Research policies, see Authors & Referees and the Editorial Policy Checklist .

Statistics

For all statistical analyses, confirm that the following items are present in the figure legend, table legend, main text, or Methods section.

n/a	Confirmed	
☐	☒	The exact sample size (*n*) for each experimental group/condition, given as a discrete number and unit of measurement
☐	☒	A statement on whether measurements were taken from distinct samples or whether the same sample was measured repeatedly
☐	☒	The statistical test(s) used AND whether they are one- or two-sided<br>*Only common tests should be described solely by name; describe more complex techniques in the Methods section.*
☒	☐	A description of all covariates tested
☒	☐	A description of any assumptions or corrections, such as tests of normality and adjustment for multiple comparisons
☐	☒	A full description of the statistical parameters including central tendency (e.g. means) or other basic estimates (e.g. regression coefficient) AND variation (e.g. standard deviation) or associated estimates of uncertainty (e.g. confidence intervals)
☐	☒	For null hypothesis testing, the test statistic (e.g. *F*, *t*, *r*) with confidence intervals, effect sizes, degrees of freedom and *P* value noted<br>*Give P values as exact values whenever suitable.*
☒	☐	For Bayesian analysis, information on the choice of priors and Markov chain Monte Carlo settings
☒	☐	For hierarchical and complex designs, identification of the appropriate level for tests and full reporting of outcomes
☒	☐	Estimates of effect sizes (e.g. Cohen's *d*, Pearson's *r*), indicating how they were calculated

Our web collection on statistics for biologists contains articles on many of the points above.

Software and code

Policy information about availability of computer code

Data collection	1. MASCOT v.2.6.2 search engine in Proteome Discoverer v2.1.1.21 and v2.2.0.388 was used to obtain spectral peaks.<br>2. Gels and blots were imaged using ChemiDoc™ Touch (Bio-RAD), Quantity One v.4.6.9, imageLab™ v6.0.0, Doc-It'LS Image acquisition software (UVP) v6.7.1 and Epson Scan (Seiko Epson) v5.1.1.0<br>3. Microscopy images were acquired using Zeiss Zen Blue v2.3 and Zen Black v2.3<br>4. Real-time PCR data was acquired using CFX Manager v3.1 (BioRad) and StepOne™ v2.3 (Applied Biosystems)
Data analysis	1. Homology search using HmmerWeb v2.41.1<br>2. Secondary structure prediction using Jpred v4<br>3. Tertiary structure prediction using HHpred v57c8707149031cc9f8edceba362c71a3762bdbf8 and Phyre v2.0<br>4. Protein sequences were aligned using T-COFFEE v11.0 and visualized using Jalview v2.11.1.0<br>5. Microscopy image processing using ImageJ v. 1.51 and Zeiss Zen Blue version v2.3<br>6. To visualize MS/MS proteomics experiments Scaffold™ v4.10.0 was used<br>7. Image Lab™ v6.0.0 and ImageJ v1.51 was used to visualize gels and blots<br>8. Data analysis was performed using Microscoft Excel v2008 and GraphPad Prism™v5.00 or v7.0a<br>9. The probability of protein disorder was predicted using IUPred2 vA<br>10. pI of the amino acid residues was predicted using ProtParam.

For manuscripts utilizing custom algorithms or software that are central to the research but not yet described in published literature, software must be made available to editors/reviewers. We strongly encourage code deposition in a community repository (e.g. GitHub). See the Nature Research guidelines for submitting code & software for further information.

Data

Policy information about availability of data

All manuscripts must include a data availability statement. This statement should provide the following information, where applicable:

- Accession codes, unique identifiers, or web links for publicly available datasets
- A list of figures that have associated raw data
- A description of any restrictions on data availability

The source data underlying Fig. 1c; Fig2a, c-f; 3b, c, e; Fig. 4a, b, f; Fig. 5d, e; Fig. 6a, b; Fig. 7c, d, g, h; Supplementary Fig. 1a-c, h; Supplementary Fig. 2a, b, d, e, g ; Supplementary Fig. 3a-j, Supplementary Fig. 4a, c; Supplementary Fig. 5c, e, f, Supplementary Fig. 6a-d, f, h and Supplementary Fig.7c-i are provided in the Source Data file. All MS proteomics data that are presented in this study have been deposited to the ProteomeXchange Consortium via jPOST with the dataset identifiers "PXD021072 (http://dx.doi.org/10.6019/PXD021072)" and "JPST000947 (https://repository.jpostdb.org/entry/JPST000947)", respectively. Publicly available databases used in the study include C. neoformans H99 proteome database (https://www.uniprot.org/proteomes/UP000010091) and Uniprot proteome database (https://www.uniprot.org/proteomes/).
Yeast strains and other data that supports the findings of this study are available upon reasonable request from the corresponding authors.

Field-specific reporting

Please select the one below that is the best fit for your research. If you are not sure, read the appropriate sections before making your selection.

☒ Life sciences ☐ Behavioural & social sciences ☐ Ecological, evolutionary & environmental sciences

For a reference copy of the document with all sections, see nature.com/documents/nr-reporting-summary-flat.pdf

Life sciences study design

All studies must disclose on these points even when the disclosure is negative.

Sample size	No statistical methods were used to predetermine sample size. Sample size and number of replicates were chosen based on other studies with similar methodologies and stated in each figure legend (PMID: 28939613, 30174190, 22561345, 30420662, 31649151 and 22561346). 100 metaphase or anaphase kinetochore clusters from each of three independent experiments was quantified to estimate kinetochore signal intensities. Where statistical analysis was applied, three independent replicates were carried out.
Data exclusions	No data was excluded from the analysis.
Replication	All the experiments were reliably reproduced, the number of independent experiments were specified in the figure legends.
Randomization	Strains and conditions that were directly compared were typically cultured together microscopy image acquisition was performed randomly. All samples were allotted randomly into experimental groups. Further randomization was not applicable.
Blinding	Investigators were not blinded. Blinding was technically difficult because experiments and analysis was carried out by the same investigators.

Reporting for specific materials, systems and methods

We require information from authors about some types of materials, experimental systems and methods used in many studies. Here, indicate whether each material, system or method listed is relevant to your study. If you are not sure if a list item applies to your research, read the appropriate section before selecting a response.

Materials & experimental systems

n/a	Involved in the study	
☐	☒	Antibodies
☒	☐	Eukaryotic cell lines
☒	☐	Palaeontology
☒	☐	Animals and other organisms
☒	☐	Human research participants
☒	☐	Clinical data

Methods

n/a	Involved in the study	
☒	☐	ChIP-seq
☒	☐	Flow cytometry
☒	☐	MRI-based neuroimaging

Antibodies

Antibodies used	1. anti-H3K9me2 antibody (Abcam, #ab1220, 1:2000)<br>2. anti-PSTAIR antibody (Abcam, #10345), 1:5000)<br>3. anti-GFP antibody (Roche, #11814460001, 1:3000)

4. anti-H4 antibody (Hayashi-Takanaka, Y. et al., 2015, #CMA400, 1:5000)
5. anti-FLAG antibody (Sigma-Aldrich, #F3165, 1:5000)
6. anti-pan-H3 antibody (Kimura, H. et al., 2008, #140-1G1, 1:3000)
7. anti-α-tubulin antibody (Sigma-Aldrich, #T9026, 1:5000)
8. anti-mouse HRP-conjugated antibody (Bangalore genei, #HO06, 1:10000)
9. anti-mouse HRP-conjugated antibody (Jackson ImmunoResearch, #315-035-003, 1:15000)
10. anti-rat HRP-conjugated antibody (Jackson ImmunoResearch, #112-035-003, 1:15000)
11. Sheep anti-mouse IgG coated uniformly onto superparamagnetic polystyrene Dynabeads (ThermoFischer Scientific, #11031)

Validation

1. anti-H3K9me2 antibody (Abcam, #ab1220): The specificity of the H3K9me2 antibody in western blot was performed by Abcam in HeLa whole cell lysate, Calf thymus histone lysate, HEK-293 lysate, and Fruit fly embryo tissue lysate.
2. anti-PSTAIR antibody (Abcam, #10345): The specificity of PSTAIR antibody in western was determined by indirect blotting using COS-7 cell extract by Abcam.
3. anti-GFP antibody (Roche, #11814460001): The specificity of GFP antibody in western blot was determined by Roche and in PMID:21803848.
4. anti-H4 antibody (Hayashi-Takanaka, Y. et al., 2015, #CMA400): The specificity of the H4 antibody is described in Hayashi-Takanaka, Y. et al., 2015.
5. anti-FLAG antibody (Sigma-Aldrich, #F3165): The specificity of FLAG antibody in immunoblotting was determined by Sigma-Aldrich.
6. anti-pan-H3 antibody (Kimura, H. et al., 2008, #140-1G1): The specificity of the pan-H3 antibody is described in Kimura, H. et al., 2008.
7. anti-α-tubulin antibody (Sigma-Aldrich, #T9026): The specificity of α-tubulin antibody in immunoblotting was determined by Sigma-Aldrich in several cultured cell lines.
8. anti-mouse HRP-conjugated antibody (Bangalore genei, #HO06): The specificity of the antibody was determined by Bangalore genei and in PMID: 30763303.
9. anti-mouse HRP-conjugated antibody (Jackson ImmunoResearch, #315-035-003): Antibody specificity was tested by Jackson ImmunoResearch by immunoelectrophoresis and/or ELISA. The antibody was found to react with whole molecule mouse IgG. It also reacts with the light chains of other mouse immunoglobulins. No antibody was detected against non-immunoglobulin serum proteins
10. anti-rat HRP-conjugated antibody (Jackson ImmunoResearch, #112-035-003): Antibody specificity was tested by Jackson ImmunoResearch by immunoelectrophoresis and/or ELISA. The antibody was found to react with whole molecule rat IgG. It also reacts with the light chains of other rat immunoglobulins. No antibody was detected against non-immunoglobulin serum proteins.
11. Sheep anti-mouse IgG coated uniformly onto superparamagnetic polystyrene Dynabeads (ThermoFischer Scientific, #11202D): The antibody reactivity to both heavy and light chains of mouse IgG1, IgG2a and IgG2b was tested by ThermoFischer Scientific.
